# Patterns of organic dysfunction in severe sepsis and septic shock patients admitted to the ICU from the emergency department: a 4-year retrospective cohort

**DOI:** 10.1186/cc14711

**Published:** 2015-09-28

**Authors:** Alan Felipe Sakai, Leonardo Lima Rocha, Camila Menezes Souza Pessoa, Thiago Domingos Corrêa, Murillo Santucci César de Assunção

**Affiliations:** 1 Intensive Care Unit, Hospital Israelita Albert Einstein, São Paulo, SP, Brazil

## Introduction

Mortality rates for severe sepsis and septic shock are decreasing through the years worldwide. Most of this improvement in mortality is associated with protocols for early recognition, resuscitation and adequate initial antibiotic choice in patients presenting with sepsis to the emergency department.

## Objective

To assess the pattern of organic dysfunction as described by the Sequential Organ Failure Assessment (SOFA) score in a 4-year period.

## Methods

Retrospective cohort study that included all clinical patients admitted to the ICU from the emergency department with diagnosis of severe sepsis or septic shock. Surgical patients and patients admitted from the ward or referrals were excluded. Demographic data and SOFA score (including individual organic dysfunctions and the general score, which is the sum of organ-specific scores) were collected from January 2008 to December 2012 at admission, 48 hours and 7 days.

## Results

We included a total of 472 patients. The median age was 70 (IQR 55-82) and most patients were male (61.2 %). Respiratory tract infection was the most common infection site (52.7 %). The median APACHE II score was 19 (IQR 17-24) and the median SOFA score at admission was 4 (IQR 3-7). A total of 58.5 % of the patients presented with severe sepsis. The SOFA score at admission was statistically significant throughout the years (*P *= 0.004). This was mainly due to circulatory and respiratory dysfunctions at admission, which significantly reduced throughout the years (*P *<0.001 and 0.045, respectively). The SOFA score significantly decreased along the days (from admission to 7 days) (*P *<0.0001) and this was consistent throughout the years.

## Conclusion

Higher severity SOFA scores are decreasing through the years probably due to the increase in early recognition and resuscitation of severe sepsis and septic shock patients admitted from the emergency department.

